# Supplementary material for: Evaluation of cattle farmers’ knowledge, attitudes, and practices regarding antimicrobial use and antimicrobial resistance in Rwanda
Source: PLoS One. 2024 Apr 11;19(4):e0300742. doi: 10.1371/journal.pone.0300742 (PMC11008905; doi:10.1371/journal.pone.0300742)
Supplement: S1 Table — (DOCX) [file pone.0300742.s001.docx]

**S1 Table:** Study Questionnaire Tool for the evaluation of knowledge, attitude, and practices towards antimicrobial use and antimicrobial resistance in Rwanda

| **Participant's Demographics and Farm's information** |
| --- |
| ID |
| Age |
| Gender |
| Education level |
| Marital status |
| Socioeconomic status |
| Role of the participant in the farm |
| How many cattle do you own? |
| Number of livestock species other than cattle |
| In the last 6 months, how many times did a veterinarian or para-veterinarian visit your farm? |
| Which diseases are the most common in your farm? |
| District |
| Sector |
| Cell |
| GPS location |
| **Biosecurity** |
| Is water available at your farm |
| Where do you get the water you use in the farm from? |
| How long do you travel to reach a water source from your farm or stable |
| How many times per day do you clean your stable |
| What do you normally use to clean your stable |
| Do you wash your hands before going in contact with cattle |
| What do you normally use to clean your hands |
| How many times do you clean your cattle per week |
| Where do you put the cow dung? |
| Does your farm have a fence |
| Do your cows graze outside of the farm |
| Does your farm have a foot bath at the entrance? |
| Which vaccines were given to your cattle? |
| **Antimicrobial Use** |
| Have you ever used antibiotics? |
| Who normally prescribes the antibiotics |
| Who normally administer the antibiotics |
| How many times have you used the antibiotics in the past 12 months? |
| What is the reason for the antibiotics use? |
| Do you give antibiotics to your cattle to promote their growth? |
| Do you give antibiotics to your cattle to prevent diseases? |
| What is the most common veterinary medicine you use in your farm? |
| Knowledge related questions |
| antibiotics are food supplement for livestock |
| antibiotics are medicine for livestock |
| antibiotics are both medicine and food supplement |
| Can antibiotics be useful for weight gain in animals? |
| Have you used antibiotics to promote growth in your cattle? |
| Can antibiotics be useful for tick borne diseases? |
| Can antibiotics show side effects after use? |
| Have you ever heard of antibiotics resistance? |
| From where did you hear it from? |
| What do you think are impacts of antibiotics resistance? |
| Can bacteria develop resistance to antibiotics? |
| Humans can have the same pathogen causing diseases as animals |
| What do you think are modes of transmission for resistant pathogens from animals to humans? |
| Attitudes related questions |
| antibiotics resistance is a public health concern |
| If the cattle is sick and the veterinarian gives them medication and they don’t get better, I should treat them myself |
| Withdrawal period should be observed to avoid antibiotics residues in food products |
| antibiotics should be prescribed by veterinarians only |
| Misuse of antibiotics can cause antibiotics resistance |
| Use of antibiotics for a long period can cause antibiotics resistance |
| Sales and distribution of antibiotics would be beneficial to reduce antibiotics resistance |
| Enhanced control and restrictions of antibiotics use would reduce emergence of antibiotics resistance |
| Antibiotics should only be used to treat animal diseases |
| Practices related questions |
| I call a veterinarian when my cattle is sick |
| I ask a friend for advise on which antibiotics to use when my cattle is sick |
| I get antibiotics from a veterinary pharmacy |
| I get antibiotics from a veterinary technician |
| I get my antibiotics from a friend and neighbors |
| I treat my cattle using antibiotics until they get better |
| I treat my cattle using antibiotics until their course of antibiotics is complete |
| When my cattle is better, I keep the remaining antibiotics for future use |
| When I have one sick cattle, I share the antibiotics to not sick animals |
| What do you do when the cow that was being treated with antibiotics dies? |
| When your cow is on antibiotics do you milk it? |
| When you milk it, what do you do with the milk? |
| Possible intervention |
| What do you think are possible measures to reduce the risk of antibiotics resistance? |
