# Supplementary material for: Evaluation of cattle farmers’ knowledge, attitudes, and practices regarding antimicrobial use and antimicrobial resistance in Rwanda
Source: PLoS One. 2024 Apr 11;19(4):e0300742. doi: 10.1371/journal.pone.0300742 (PMC11008905; doi:10.1371/journal.pone.0300742)
Supplement: Attachment — Submitted filename: Response to reviewers - PONE-D-23-36366.docx [file pone.0300742.s008.docx]

# **Review PLOS One - PONE-D-23-36366**

We want to thank the two reviewers and the academic Editor for the very helpful and constructive comments. Please find our responses to your comments below.

## **Responses to reviewers' comments**

**Reviewer #1**

**Comment 1:** This paper is well written, and the topic is relevant.

**Response:** We are sincerely grateful for your time taken to read and evaluate our paper. We believe that your comments and suggestions will ensure the paper’s scientific content is sound and accurate.

**Comment 2:** The use of the mean KAP score as a cutoff to differentiate good and bad, can be difficult for future reference. It can be logical for this specific study but overall difficult to make comparisons with other similar studies.

**Response:** Thank you for this comment. This study extensively assessed cattle farmers' knowledge, attitudes, and practices towards antimicrobial use and antimicrobial resistance in the eastern province of Rwanda. There is a lack of similar studies in Rwanda; consequently, there was no prior knowledge of how the study participants would perform during the assessment. Given that there is no universally accepted cut-off for knowledge, attitudes and practices [1], a relative cut-off is indicated to allow comparison within the studied group.

However, using a relative cut-off doesn’t prevent comparisons with similar studies. The current research has generated percentage scores for the individual and overall performance of the study participants. Hence, other scientists could compare their total percentage scores to the scores reported in this study.

**Comment 3:** Table 2: Second and third items in the first column are interchanged, correction is needed. “How long do you travel to reach a water source from your farm or stable”, need to be the second and “Where do you get the water you use in the farm from?”, the third point.

**Reviewer #2**

**Comment 1:** The study appears to be well conducted, with an appropriate sample size, and executed correctly. However, it is recommended to order the methodology from general to particular, and in each part, provide as many details as possible.

**Response:** We would like to take this opportunity to sincerely thank the reviewer for a thorough assessment of our manuscript. We are confident that the suggestions will strengthen the paper’s content and clarity.

We have revised the methodology section to ensure that the content is ordered from general to particular in a way that enhances the reader's understanding.

We have also added details to ensure the analysis is understood and transparent to the readership.

**Comment 2:** The statistical analysis is simple and appears to have been carried out well. However, it is essential that the statistical analysis be explained in the methodology and not in the results. In addition, the assumptions of the analyses and the names of the procedures in SAS need to be detailed.

**Response:** Thank you for this insightful comment. Details on how the data analysis was rewritten are provided below on a similar comment.

**Comment 3:** Finally, the manuscript presents various but small errors in the writing, so it is recommended to review. Some comments are found in the following lines.

**Response:** We are grateful for your report about the observed errors and typos. We have reviewed the manuscript to ensure there are no further errors.

Specifically, the reported typos and errors in the abstract, methods, results, discussion, and conclusion sections were addressed per the reviewer’s recommendations.

***Abstract and Introduction***

**Comment 4:** Line 38. It is suggested not to use acronyms in keywords. Additionally, Antimicrobial resistance and Antimicrobial Use are already in the keyword list. I also suggest changing the country in the keywords and using a word that contextualizes your work, such as cattle farming.

**Response:** The acronyms were removed as well as the country in the keywords. We have added “Cattle Farming” and “Small-scale farms” to include a keyword related to the context of the study.

**Comment 5:** Line 68. I suggest rewriting the phrase "as well as in food animals and their products" to "and food animals and their products."

**Response:** “as well as” was removed and replaced by “and”. Hence the sentence reads as “Resistance has been detected in humans [2] and food animals and their products [3–5].

***Methods***

**Comment 6:** Lines 77 to 85. In the Study Setting, I suggest being concise in saying where the study will be carried out and the characteristics of choosing those places rather than providing data on the organization of the country.

**Response:** These lines were rewritten to remove the country’s organization while keeping the characteristics of the study area.

**Comment 7:** Lines 87-88. I suggest writing: "Map of the study area with the interviewed farm locations (red points) in the Kayonza, Gatsibo, and Nyagatare districts."

**Response:** The figure 1 title has been changed as per the recommendation.

It now reads as follows: ***“Map of the study area with the interviewed farm locations (red points) in the Kayonza, Gatsibo, and Nyagatare districts”***

**Comment 8:** Line 93: Study population and sampling strategy: In which statistic program was the calculation of the sample of interviews carried out? It should be clarified.

**Response:** The sample calculation was conducted using the Cochrane formula indicated for categorical variables [6]. A short sentence was added to have this clarified.

**Comment 9:** Line 96: I suggest changing "with reference to" to "concerning."

**Response:** To avoid changing the meaning of the sentence, we have modified “with reference to” to “in line with”.

**Comment 10:** 103-104: I suggest changing to "Finally, Excel's RAND function randomly selected three cells per sector (18 cells) and three villages per cell (54 villages)."

**Response:** The sentence was modified as recommended.

**Comment 11:** 107-127: It is preferable to summarize the six items of the survey in tables.

**Response:** The questionnaire (S1 Table) summarizes the 6 items of the survey. We have already submitted the study questionnaire as part of the supplementary materials. To improve the reader’s experience, we have added an in-text citation (S1 table) that will direct the reader to the questionnaire if clicked on.

**Comment 12:** 109-110: I suggest editing "The research team designed and reviewed the data collection tool internally for content, understanding, and relevance."

**Response:** Thank you for this suggestion. We have edited as recommended.

**Comment 13:** 124: The sentence "This entailed questions on how the interviewees felt or thought about topics related to AMR" generates confusion, so I suggest removing

**Response:** The sentence was removed.

**Comment 14:** 129: I think that "Data collection procedure" should be included at the beginning of "Data collection tool and validation" since it provides the context of the data collection. Then, you can continue with the survey's structure.

**Response:** The content under data collection and procedure was moved in the previous sub-section (data collection tool and validation).

**Comment 15:** 140-157: I consider it important, after establishing in which statistical program the work was carried out, to mention the SAS procedures or packages that were used to obtain the results (data summary, associations, etc.). On the other hand, I consider that the description of analyses that were carried out should be described in the same order in which the results are presented. For example, descriptive statistics are mentioned near the end of the statistical analysis, but it is the first result. On the other hand, line 299 mentions a logistic regression analysis, which should be explained in detail in data analysis, including the procedure used in SAS and dependent and independent variables. The same goes for Pearson on line 268.

**Response:** Thank you for this constructive comment. The analyses were carried out using SPSS software with a user-friendly graphical interface. Usually, data analysis in SPSS is done by clicking a drop-down menu that allows the user to select an appropriate test. For example, (1) descriptive statistics is done by clicking on “analyze”, then “descriptives”, and finally “descriptive statistics. (2) chi-square can be done by clicking on “analyze”, then “descriptives” and finally “crosstabs”. Finally, the binary logistic regression can be conducted by clicking on “analyze”, then “regression” and lastly “binary logistic. These details are often fundamental to any SPSS user and could be found in the tutorials widely available for the software.

We have modified the data analysis paragraph to include more details about the analysis as recommended. The paragraph now reads as follows:

*“Data analyses were performed using SPSS Statistics version 27 (IBM Corp., Armonk, NY, USA) graphical user interface.*

*The demographics, knowledge levels, practice levels, attitude levels, and biosecurity-related items were summarized as counts and percentages using descriptive statistics.*

*The demographic characteristics included age category, gender, education level, socioeconomic status, marital status, district, participant role at the visited farms and the number of cattle owned.*

*Biosecurity-related items were viewed independently to create variables whose frequencies were calculated in the data analysis.*

*The three dependent variables were knowledge, attitudes, and practice (KAP) levels. Knowledge and practices were graded one point if correct and zero if incorrect, whereas, for attitudes, the correct answer was graded as 2 points, neutral as 1 point, and incorrect answers were 0, based on the validated rubric. The total grades per participant were used to calculate the mean KAP score, which was used as a cutoff for “good” and “poor” knowledge, attitude, or practice. The calculated means were 69%, 47%, and 50% for knowledge, attitude, and practice, respectively. Participants with scores above or equal to the cutoff were considered to have good knowledge, attitude, or practice. In contrast, those with scores below the cutoff were considered to have poor knowledge, attitude, or practice.*

*Associations between demographics and KAP levels were analyzed using a chi-square test of association between KAP variables (Knowledge, attitudes, and practice levels) and potential explanatory independent variables (the demographic characteristics). The demographic factors that showed a significant association during the chi-square univariate analysis were used to perform a multivariable binary logistic regression analysis to identify the key independent variables affecting KAP toward AMR in the study area. The 5% level of significance was used to interpret the association results. The 95% confidence intervals for the adjusted odds ratios were used to evaluate the significance and direction of the associations.”*

***Results***

**Comment 16:** In the results section, it is essential to explain in the text what all the tables contain before describing them. For example, on line 160, it would be essential to write: “The socio-demographic data is summarized in Table 1” and then write the description of the Table.

**Response:** Thanks for these recommendations. We added a short sentence to introduce each table before further description of the results included in the table.

**Comment 17:** Another comment is that “Figure (Fig.)” is summarized, but not the word Table, so I suggest keeping both without abbreviation for the entire text.

Response: We appreciate the reviewer’s suggestion. The figure is abbreviated according to PLOS ONE guidelines on figure captions as detailed on the following link <https://journals.plos.org/plosone/s/figures#loc-captions>.

**Comment 18:** 165: Correct the title of Table 1: Socio-demographics (data or variables) of cattle farmers

Response: The title has been changed as recommended. The title reads as follows: “Socio-demographic data of cattle farmers”

**Comment 19:** In Tables 1 and 2, it is important to be clear in the titles of each column. There must be a header in the columns where the response levels are described. In the column where the number of farmers per level and their percentage are described, it must also have a header.

**Response:** Thank you for the comment. We have included the headers on table 1, 2 and 3 as recommended.

***Discussion***

**Comment 20:** 343-344: change "use of" to just "use".

**Response:** We are thankful for the reviewer’s suggestion. The sentence discusses the variance in cattle producers’ use **of veterinary services**. Hence, changing “use of” to “use” would alter the sentence’s meaning.

**Comment 21:** 351-152: Before these lines, it is stated that AMU is due to different diseases. Then biosafety with the AMU is mentioned. It would be important to make the link between diseases, lack of biosecurity, and AMU here.

**Response:** We have added this sentence to make the suggested link between AMU, biosecurity, and diseases:

“*Biosecurity measures include practices implemented on farms to prevent the introduction and circulation of pathogens. Consequently, implementing biosecurity measures is known to reduce the occurrence of infectious diseases on farms and could potentially minimize antimicrobials [7].*”

**Comment 22:** 394-395: Which studies?

**Response:** We have added relevant references to clarify the studies we were talking about. Hence, the sentence reads as follows:

“*This is higher than levels found in similar studies conducted in Ethiopia [1,8]*.”

**Comment 23:** 420-423: It is important that when reference is made to other studies, the context of those studies is provided (For example, place and type of rancher)

**Response:** We thank the reviewer for this insight. The sentence was modified, and it now reads as follows: “*Extensive antibiotic misuse may enhance selection pressure for the formation of AMR and have detrimental health and economic consequences. This was confirmed by a study conducted in Ghana, Kenya, Tanzania, Zambia and Zimbabwe on households and pastoralists keeping layers, cattle, sheep, and goats [9].*”

***Conclusion***

**Comment 24:** 456-457: In the sentence “Our findings showed a considerable discrepancy in the KAP levels of farmers in terms of gender, education level, district, and other sociodemographic characteristics,” it would be necessary to mention those discrepancies briefly.

Response: The sentence was modified to make it clearer. Now the paragraph reads as follow: *“Our findings showed considerable differences in the KAP levels of participating farmers. For instance, gender, education level, and the district correlated with the level of knowledge, attitudes, or practices. They also highlighted improper behavior around AMU, such as farmers getting antimicrobials from friends and neighbors without any information on dosage or proper use from a veterinarian. These findings underscore the need for interventions to close gaps in the farmers’ KAP of AMU and AMR while considering the different disparities among farmers in the study area. Therefore, AMR stewardship should entail a behavioral change to tackle the differences between knowledge, attitudes, and practices”.*

***Comment 25:*** 464-469: In my opinion, I think that this part of the conclusion escapes the proposed objectives

**Response:** We have rephrased this section to provide the readers with our reflection on the “so what” implications of our findings.

**References:**

1.	Geta K, Kibret M. Knowledge, attitudes and practices of animal farm owners/workers on antibiotic use and resistance in Amhara region, north western Ethiopia. Sci Rep. 2021;11: 21211. doi:10.1038/s41598-021-00617-8

2.	Ntirenganya C, Manzi O, Muvunyi CM, Ogbuagu O. High prevalence of antimicrobial resistance among common bacterial isolates in a tertiary healthcare facility in Rwanda. Am J Trop Med Hyg. 2015;92: 865–870. doi:10.4269/ajtmh.14-0607

3.	Ndahetuye JB, Twambazimana J, Nyman A-K, Karege C, Tukei M, Ongol MP, et al. A cross sectional study of prevalence and risk factors associated with subclinical mastitis and intramammary infections, in dairy herds linked to milk collection centers in Rwanda. Preventive Veterinary Medicine. 2020;179: 105007. doi:10.1016/j.prevetmed.2020.105007

4.	Ndahetuye JB, Artursson K, Båge R, Ingabire A, Karege C, Djangwani J, et al. MILK Symposium review: Microbiological quality and safety of milk from farm to milk collection centers in Rwanda*. Journal of Dairy Science. 2020;103: 9730–9739. doi:10.3168/jds.2020-18302

5.	Manishimwe R, Moncada PM, Musanayire V, Shyaka A, Scott HM, Loneragan GH. Antibiotic-Resistant Escherichia coli and Salmonella from the Feces of Food Animals in the East Province of Rwanda. Animals (Basel). 2021;11: 1013. doi:10.3390/ani11041013

6.	Bartlett JE, Kotrlik JW, Higgins CC. Organizational Research: Determining Appropriate Sample Size in Survey Research. Information Technology, Learning, and Performance Journal. 2001;19.

7.	Robertson ID. Disease Control, Prevention and On-Farm Biosecurity: The Role of Veterinary Epidemiology. Engineering. 2020;6: 20–25. doi:10.1016/j.eng.2019.10.004

8.	Gebeyehu DT, Bekele D, Mulate B, Gugsa G, Tintagu T. Knowledge, attitude and practice of animal producers towards antimicrobial use and antimicrobial resistance in Oromia zone, north eastern Ethiopia. PLoS One. 2021;16: e0251596. doi:10.1371/journal.pone.0251596

9.	Caudell MA, Dorado-Garcia A, Eckford S, Creese C, Byarugaba DK, Afakye K, et al. Towards a bottom-up understanding of antimicrobial use and resistance on the farm: A knowledge, attitudes, and practices survey across livestock systems in five African countries. PLOS ONE. 2020;15: e0220274. doi:10.1371/journal.pone.0220274
