# Supplementary material for: Evaluation of cattle farmers’ knowledge, attitudes, and practices regarding antimicrobial use and antimicrobial resistance in Rwanda
Source: PLoS One. 2024 Apr 11;19(4):e0300742. doi: 10.1371/journal.pone.0300742 (PMC11008905; doi:10.1371/journal.pone.0300742)
Supplement: Attachment — Submitted filename: Response to reviewers - PONE-D-23-36366_R2.docx [file pone.0300742.s009.docx]

# **Review PLOS One - PONE-D-23-36366**

We want to thank the reviewers for accepting our responses. We are enclosing below the responses to the remaining reviewer’s questions.

## **Responses to reviewers' comments**

**Reviewer #2**

**Comment 1:** Reviewer #2: I am satisfied with responses to the comments. You should avoid formulating sentences that are too short on lines 102 and 161 to 168. Also, on line 161 you must delete (&quot;)

**Response:** We are sincerely grateful for you’re the reviewer’s time to read and evaluate our paper. We believe that the final comments and suggestions will improve the clarity of our paper. We have modified the short sentences identified by the reviewer.

**Line 101:** The sentences were as follows:

To account for missing data and invalid entries, 15% of the 383 participants were added to the required sample size, rounding to 441 participants. Participants were selected using a multistage sampling method.

The two sentences were combined and to read as follows:

*To account for missing data and invalid entries, 15% of the 383 participants were added to the required sample size, rounding to 441 participants, selected using a multistage sampling method.*

***Line 161 to 168:*** The sentences were modified to remove very short sentences. The paragraph reads as follows:

*Most participants were male (61.5%; Table 1) and the majority (40.1%) were aged between 46 and 65 years and the mean age was 47.7 years (SD = 16.4). More than half (54%) of the participants had a primary school education, and the vast majority (86.4%) were farm owners who owned between 1–3 cattle (62.1%) (Table 1).*

*The* ***“*** *symbol on Line 162 was also deleted.*
